# Supplementary material for: Rosmarinic Acid and Sodium Citrate Have a Synergistic Bacteriostatic Effect against Vibrio Species by Inhibiting Iron Uptake
Source: Int J Mol Sci. 2021 Dec 1;22(23):13010. doi: 10.3390/ijms222313010 (PMC8657459; doi:10.3390/ijms222313010)
Supplement: Supplementary file 1 [file ijms-22-13010-s001.zip › ijms-1473712-supplementary.pdf]

# Supplementary information

## 1. Supplementary Experimental procedures

### 1.1 Structure solution and refinement for VmFbpA

The X-ray diffraction data were indexed, integrated, and scaled using the XDS program [1]. Initial structural models of apo VmFbpA were obtained by molecular replacement with Morlep [2] using the crystal structures of TtFbpA (PDB entry: 3WAE [3]) as the search model. Further model building and refinement were performed with REFMAC5 [4], Coot [5], and Phenix [6]. The tertiary structure was visualised with PyMOL [7].

### 1.2 Reference for Supplementary Experimental procedures


1. Kabsch, W. XDS. *Acta Crystallogr. Sect. D Biol. Crystallogr.* **2010**, *66*, 125–132, doi:10.1107/S0907444909047337.
2. Vagin, A.; Teplyakov, A. MOLREP : an automated program for molecular replacement. *J. Appl. Crystallogr.* **1997**, *30*, 1022–1025, doi:10.1107/S0021889897006766.
3. Wang, S.; Ogata, M.; Horita, S.; Ohtsuka, J.; Nagata, K.; Tanokura, M. A novel mode of ferric ion coordination by the periplasmic ferric ion-binding subunit FbpA of an ABC-type iron transporter from *Thermus thermophilus* HB8. *Acta Crystallogr. Sect. D Biol. Crystallogr.* **2014**, *70*, 196–202, doi:10.1107/S1399004713026333.
4. Murshudov, G.N.; Skubák, P.; Lebedev, A.A.; Pannu, N.S.; Steiner, R.A.; Nicholls, R.A.; Winn, M.D.; Long, F.; Vagin, A.A. REFMAC 5 for the refinement of macromolecular crystal structures. *Acta Crystallogr. Sect. D Biol. Crystallogr.* **2011**, *67*, 355–367, doi:10.1107/S0907444911001314.
5. Emsley, P.; Cowtan, K. Coot : model-building tools for molecular graphics. *Acta Crystallogr. Sect. D Biol. Crystallogr.* **2004**, *60*, 2126–2132, doi:10.1107/S0907444904019158.
6. Afonine, P. V.; Grosse-Kunstleve, R.W.; Echols, N.; Headd, J.J.; Moriarty, N.W.; Mustyakimov, M.; Terwilliger, T.C.; Urzhumtsev, A.; Zwart, P.H.; Adams, P.D. Towards automated crystallographic structure refinement with phenix.refine. *Acta Crystallogr. Sect. D Biol. Crystallogr.* **2012**, *68*, 352–367, doi:10.1107/S0907444912001308.
7. Delano, W.L. PyMOL: An open-source molecular graphics tool. *CCP4 Newsl. Protein Crystallogr* **2002**, *40*, 82–92.

## 2. Supplementary Table

**Table S1.** Crystallographic data collection and refinement statistics for VmFbpA.

| X-ray diffraction data collection (BL44XU at SPring-8) | |
|---|---|
| Wavelength (Å) | 0.899995 |
| Resolution range (Å) | 45.40-1.86 (5.54-1.86) |
| Space group | $P6_322$ |
| Unit cell parameter (Å) | |
| *a* | 90.79 |
| *b* | 90.79 |
| *c* | 149.68 |
| Number of reflections | 456245 |
| Unique reflections | 58147 |
| Completeness (%) | 99.8 (99.0) |
| Redundancy | 7.8 (6.8) |
| $R_{merge}$ (%) | 9.3 (71.8) |
| $I / \sigma (I)$ | 15.17 (2.81) |
| **Refinement statistics** | |
| $R_{work}$ (%) | 17.58 |
| $R_{free}$ (%) | 19.60 |
| RMSD | |
| Bond length (Å) | 0.0062 |
| Bond angle (°) | 0.847 |
| Number of atoms | |
| Protein | 2364 |
| Water | 238 |
| Ramachandran plot (%) | |
| Favored | 99.35 |
| Allowed | 0.65 |
| Outliers | 0.00 |

Values in parentheses are for the highest-resolution shell.

**Table S2.** Affinity between RA and VmFbpA of each docking model at the $Fe^{3+}$ binding site.

| Model No. | Affinity (Δ*G*, kcal/mol) | $K_D$ at 298.15 K (μM) |
|---|---|---|
| Model 1 | -6.8 | 10 |
| Model 2 | -6.8 | 10 |
| Model 3 | -6.8 | 10 |
| Model 4 | -6.9 | 8.4 |
| Model 5 | -6.9 | 8.4 |
| Model 6 | -7.0 | 7.1 |
| Model 7 | -7.0 | 7.1 |
| Model 8 | -7.0 | 7.1 |
| Model 9 | -7.0 | 7.1 |

**Table S3.** Interactions between VmFbpA and RA, calculated by PDBePISA

| No. | VmFbpA | Distance (Å) | RA |
|---|---|---|---|
| | | Model 1 | |
| 1 | ARG 9 [NH2] | 3.22 | RA [OAM] |
| 2 | ARG 9 [NH2] | 2.07 | RA [OAR] |
| 3 | ASN 193 [ND2] | 2.65 | RA [OAY] |
| 4 | ASN 138 [ND2] | 3.72 | RA [OAZ] |
| 5 | GLY 140 [N] | 3.78 | RA [OAZ] |
| 6 | VAL 259 [O] | 3.03 | RA [OAH] |
| | | Model 2 | |
| 1 | THR 36 [OG1] | 3.04 | RA [OAG] |
| 2 | THR 36 [N] | 2.59 | RA [OAG] |
| 3 | TYR 196 [OH] | 3.77 | RA [OAL] |
| 4 | TYR 196 [OH] | 3.18 | RA [OAQ] |
| 5 | ARG 9 [NH2] | 2.04 | RA [OAY] |
| 6 | ASP 35 [OD1] | 3.51 | RA [OAG] |
| 7 | GLY 34 [O] | 2.64 | RA [OAH] |
| 8 | GLN 58 [OE1] | 1.56 | RA [OAY] |
| | | Model 3 | |
| 1 | ASN 193[ND2] | 2.34 | RA [OAG] |
| 2 | ASN 193[ND2] | 3.50 | RA [OAH] |
| 3 | GLY 140 [N] | 3.50 | RA [OAH] |
| 4 | TYR 196 [OH] | 2.19 | RA [OAY] |
| 5 | TYR 195 [OH] | 1.88 | RA [OAG] |
| 6 | ARG 9 [NH2] | 1.75 | RA [OAL] |
| 7 | GLN 58 [OE1] | 1.59 | RA [OAL] |
| | | Model 4 | |
| 1 | ARG 9 [NH2] | 2.75 | RA [OAM] |
| 2 | ASN 193 [ND2] | 3.27 | RA [OAY] |
| 3 | TYR 196 [OH] | 2.60 | RA [OAY] |
| 4 | ASN 138 [ND2] | 3.90 | RA [OAY] |
| 5 | ASN 138 [ND2] | 3.82 | RA [OAZ] |
| 6 | GLN 58 [OE1] | 1.54 | RA [OAR] |
| | | Model 5 | |
| 1 | ARG 9 [NH2] | 3.16 | RA [OAL] |
| 2 | GLN 58 [OE1] | 1.58 | RA [OAQ] |
| 3 | ARG 100 [NE] | 1.84 | RA [OAR] |

| | | | |
|---|---|---|---|
| 4 | ARG 100 [NH2] | 1.58 | RA [OAR] |
| | | Model 6 | |
| 1 | TYR 196 [OH] | 3.06 | RA [OAQ] |
| 2 | ARG 100 [NH2] | 2.88 | RA [OAR] |
| 3 | ARG 100 [NH1] | 1.82 | RA [OAY] |
| 4 | ARG 9 [NH2] | 2.71 | RA [OAY] |
| 5 | ARG 9 [NH2] | 2.70 | RA [OAZ] |
| 6 | GLU 262 [OE2] | 2.51 | RA [OAY] |
| | | Model 7 | |
| 1 | ARG 199 [NH1] | 3.51 | RA [OAL] |
| 2 | ARG 199 [NH2] | 2.23 | RA [OAM] |
| 3 | TYR 195 [OH] | 2.16 | RA [OAY] |
| 4 | GLN 58 [O] | 3.62 | RA [OAZ] |
| 5 | ARG 199 [NH2] | 1.84 | RA [OAQ] |
| 6 | GLN 58 [NE2] | 1.81 | RA [OAZ] |
| | | Model 8 | |
| 1 | ASN 193 [ND2] | 2.60 | RA [OAG] |
| 2 | GLU 262 [OE2] | 2.39 | RA [OAY] |
| 3 | GLN 58 [O] | 3.58 | RA [OAZ] |
| 4 | TYR 195 [OH] | 1.65 | RA [OAG] |
| | | Model 9 | |
| 1 | ASN 193 [ND2] | 2.46 | RA [OAH] |
| 2 | ARG 9 [NH2] | 2.16 | RA [OAY] |
| 3 | GLN 58 [O] | 3.75 | RA [OAG] |
| 4 | GLU 262 [OE2] | 2.18 | RA [OAZ] |

**Table S4.** FbpA proteins involved in $Fe^{3+}$ uptake by Vibrio species.

| *Vibrio* species | Protein ID | Name | Length | Identity to VmFbpA | E-value | Conserved residues for RA binding |
|---|---|---|---|---|---|---|
| *V. metschnikovii* | WP_004394209.1 | VmFbpA | 332 | 100% | 0.0 | 100% |
| *V. vulnificus* | WP_130359037.1 | VvFbpA | 337 | 31.2% | 4e-46 | 57.1% |
| *V. parahaemolyticus* | KOY43573.1 | VpFbpA | 337 | 31.1% | 9e-43 | 50.0% |
| *V. cholerae* | WP_142739098.1 | VcFbpA | 337 | 31.0% | 3e-34 | 50.0% |

## 3. Supplementary Figures

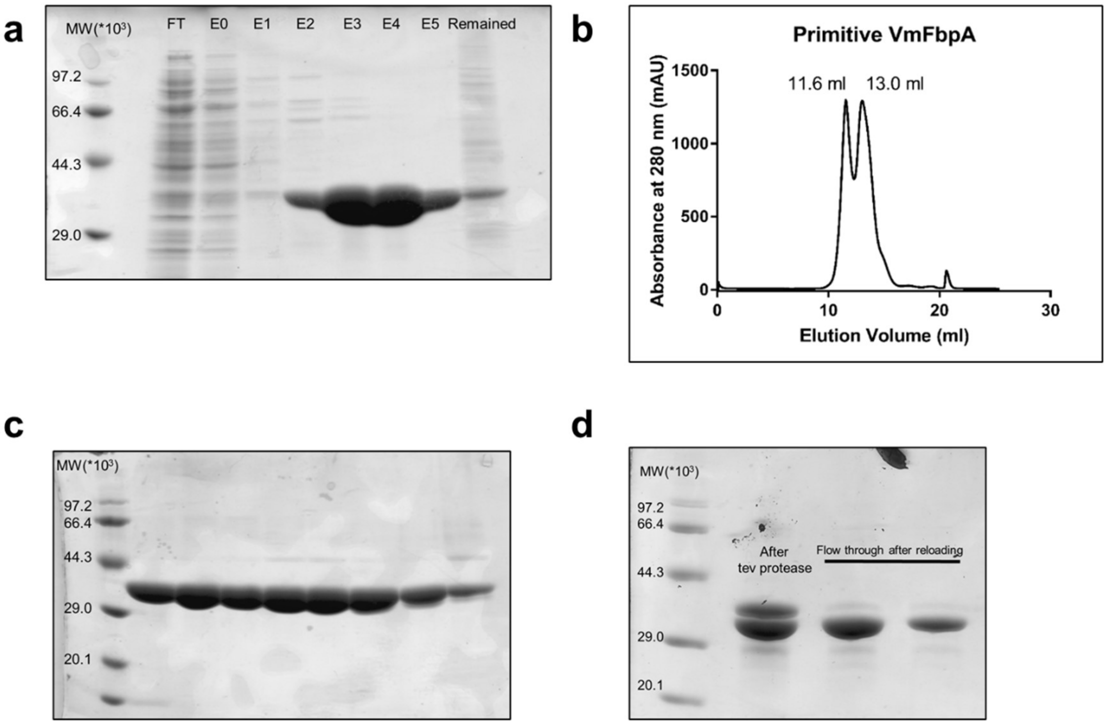


**Figure. S1.** Purification of VmFbpA.

**a**: SDS-PAGE of the Ni-NTA resin affinity chromatography fractions. The first lane on the left is the molecular weight marker. Lane FT is the flow-through after loading the Ni-NTA resin. Lane E0 is the fraction eluted by washing buffer 1. Lane E1 is the fraction eluted by washing buffer 2. Lane E2 is the fraction eluted by elution buffer 1. Lane E3 is the fraction eluted by elution buffer 2. Lane E4 is the fraction eluted by elution buffer 3. Lane E5 is the fraction eluted by elution buffer 4. Lane “Remained” is the Ni-NTA resin after elution. **b**: Gel filtration chromatography of VmFbpA collected from E2 to E5. **c**: Image of SDS-PAGE for the fractions (1 mL/fraction) from the elution peak collected from 10 mL to 17 mL. The first lane on the left is the molecular marker. **d**: SDS-PAGE to verify 6 × His-tag cleavage. The first lane on the left is the molecular marker. Lane 1 is VmFbpA incubated overnight with TEV protease. Lanes 2 and 3 are samples of the flow-through after reloading the Ni-NTA resin.

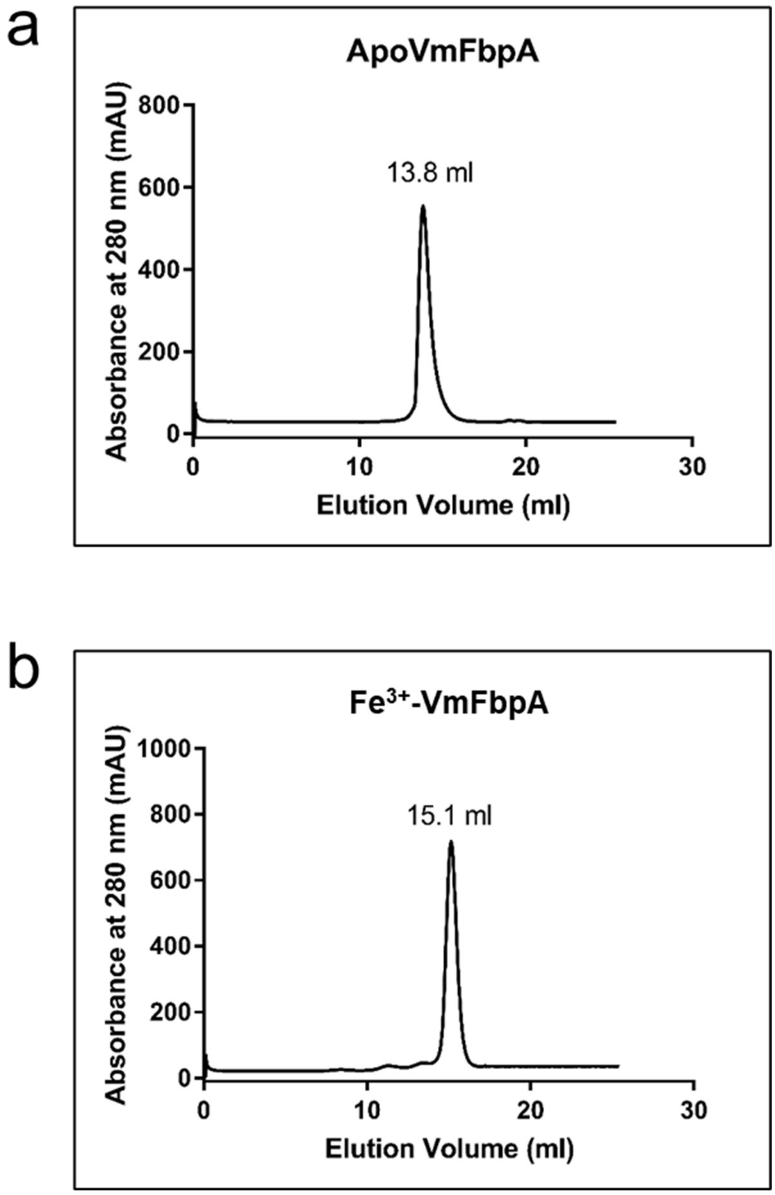


**Figure. S2.** Preparation of $Fe^{3+}$-bound VmFbpA and apo VmFbpA.

**a**: Gel filtration chromatography of apo VmFbpA. **b**: Gel filtration chromatography of $Fe^{3+}$-VmFbpA.

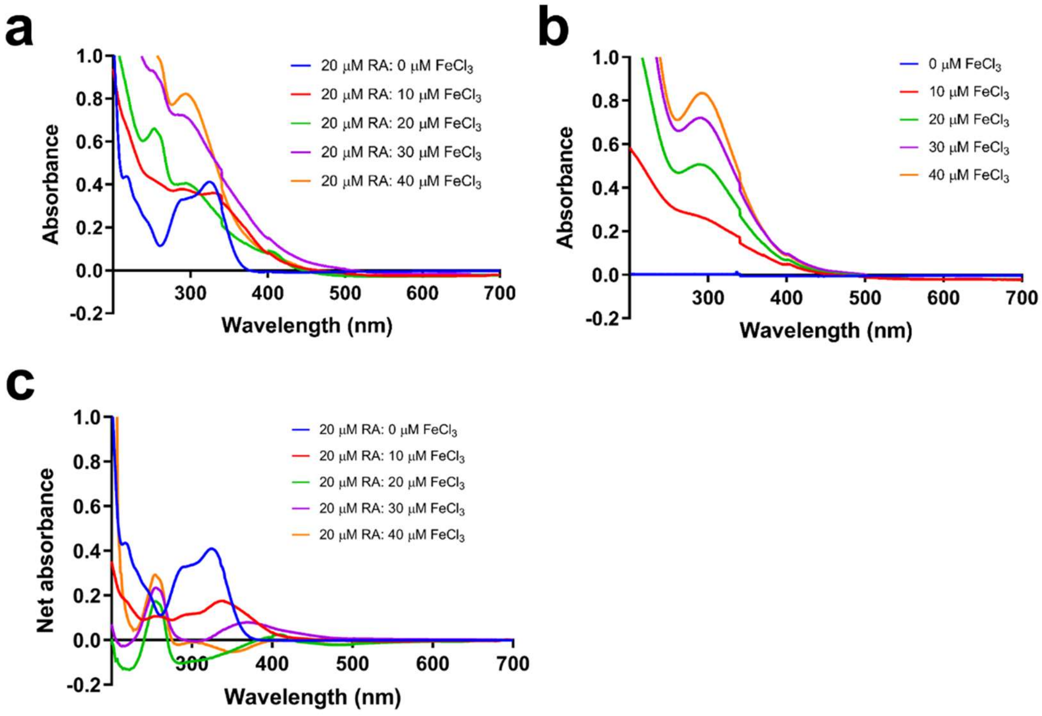


**Figure. S3.** Spectral analysis of RA with apo VmFbpA and $Fe^{3+}$.

**a**: Absorbance spectra (200–700 nm) of RA supplemented with $FeCl_3$ (0, 10, 20, 30, 40 μM). **b**: Absorbance spectra (200–700 nm) of $FeCl_3$ (0, 10, 20, 30, 40 μM) solutions. **c**: Net spectra (200–700 nm) of RA supplemented with $FeCl_3$ after subtraction of the respective $FeCl_3$ spectra.

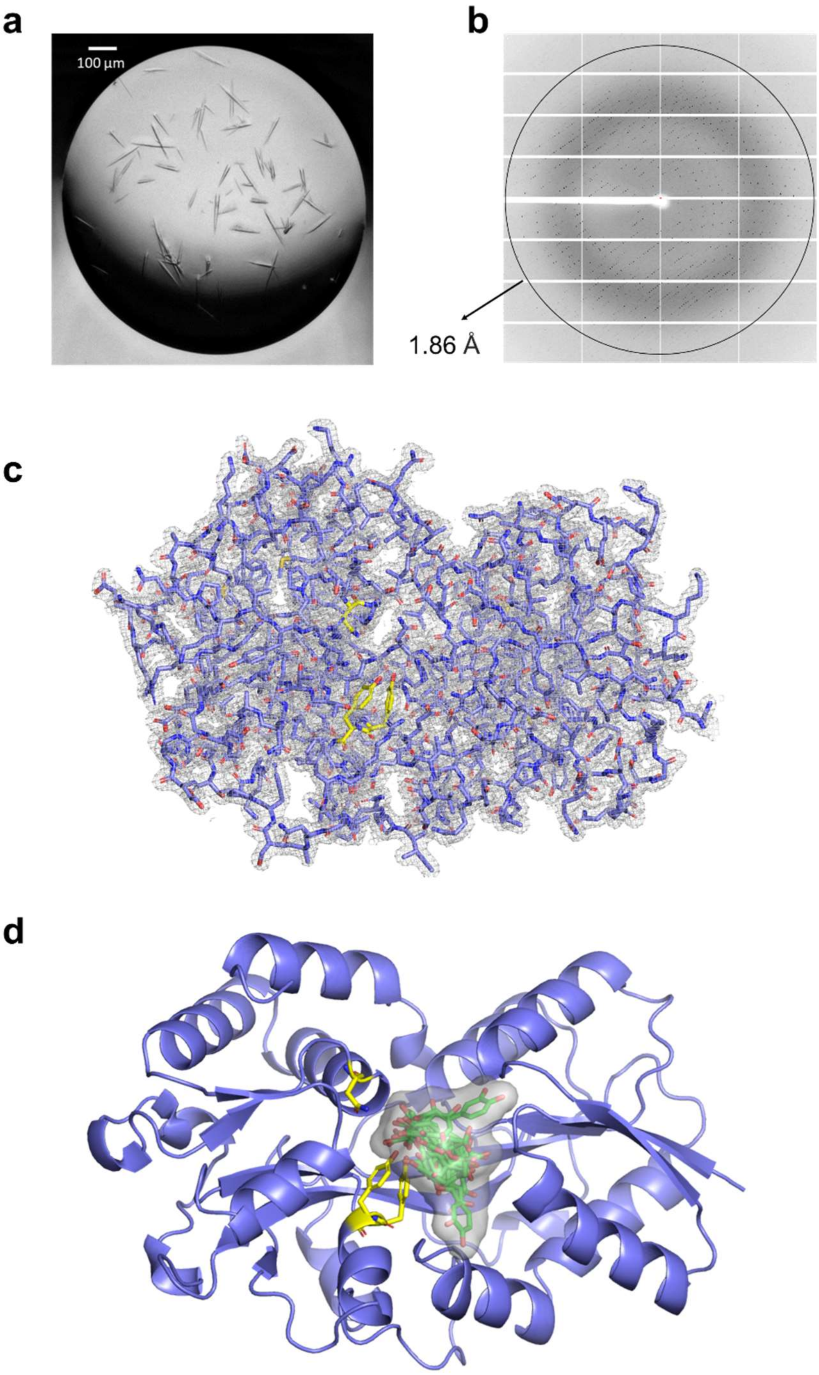


**Figure. S4.** X-ray crystallographic analysis of VmFbpA.

**a**: Protein crystals of apo VmFbpA. The crystallization conditions were as follows: 0.25 M ammonium tartrate dibasic, 25% PEG3350, 100 mM Tris-HCl (pH 7.0). **b:** X-ray diffraction pattern of an apo VmFbpA crystal. Diffraction spots were obtained from X-ray diffraction experiments using the crystals harvested in **a**. The maximum resolution was 1.8 Å. **c**: Crystal structure of apo VmFbpA as a ball-and-stick model. The structure is masked by the electron density and colored in purple. The $Fe^{3+}$ binding site (Asn138, Tyr195, Tyr196) is in yellow. **d**: Docking models of RA binding to the crystal structure of apo VmFbpA. RAs from nine docking models were overlayed in green.

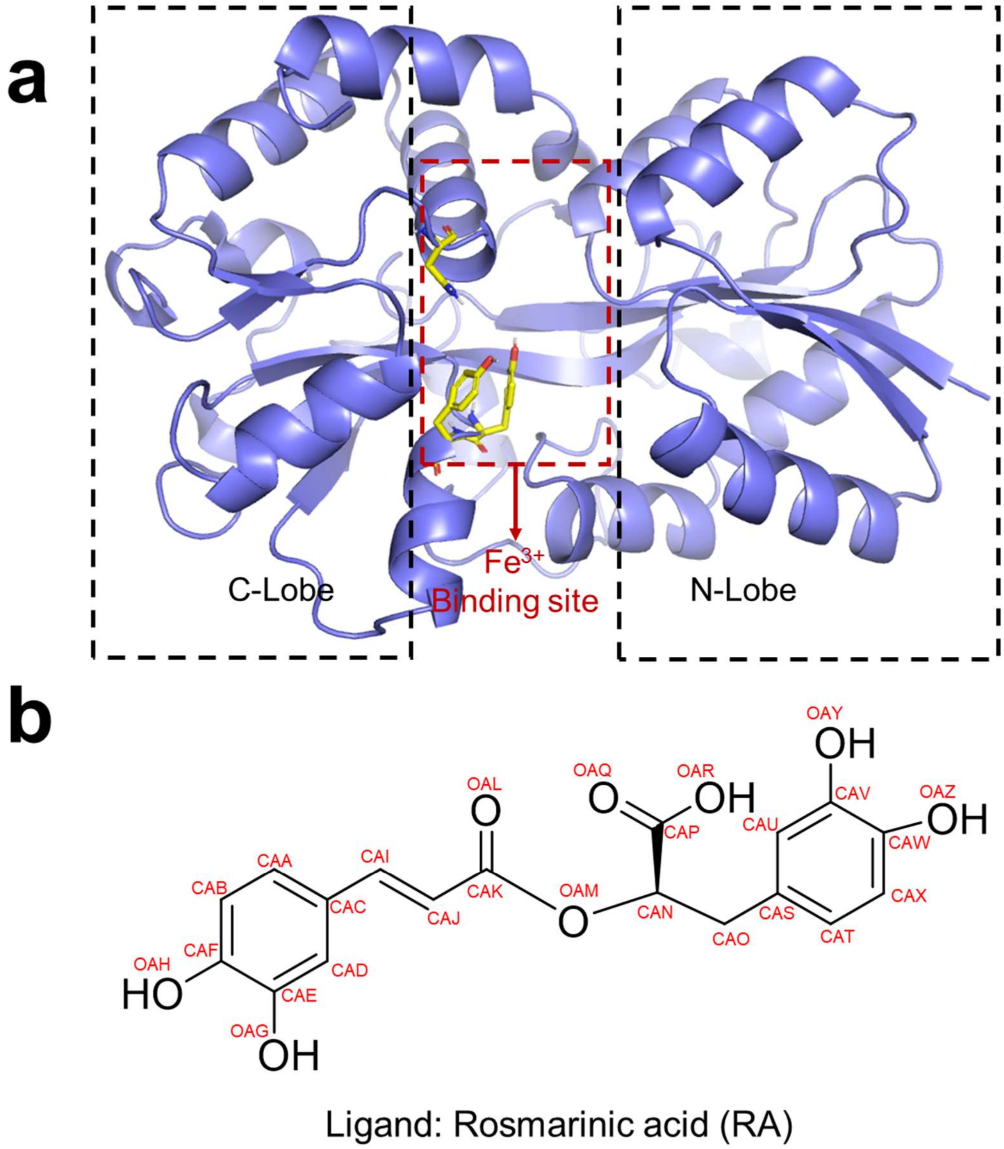


**Figure. S5.** Receptor and ligand prepared for docking.

**a**: Areas of VmFbpA for docking analysis. Three areas (C-Lobe, N-Lobe, and the $Fe^{3+}$ binding site) were chosen as potential docking areas of RA. **b**: Molecular structure of RA prepared by ChemBioDraw Ultra (version 13.0.0.3015). The red marks are the atom IDs generated by PyMOL.

```
VmFbpA    1 MQKKLLSTVALVSSLITAPLLHAQDITLYSGRGETLVKPIIEQFEKQSGIKVNVRYGDTA
VvFbpA    1 M-KKLLTLSALACATLAPTAMAAEEVNVYSYRQPFLVEPMFNEFTKETGIKVNVMFAKTG
VpFbpA    1 M-KKLLTLSALTCATLAPTAMAAEEVNVYSYRQPFLVEPMFNEFTKETGIKVNVKFAKKG

VmFbpA   61 QLAVLLQEEGARSPADVYWGQDAGAMGALANAGLLATLP-EAVYKQLPEIYTSKTGQWVA
VvFbpA   60 -IAEKLAQEGEYSPADVILTVDISRLAELTDKGLVQPVNSEIIEANVPAQYQDKENEWFA
VpFbpA   60 -LAEKLAQEGEYSPADVILTTDISRLAELTNKGLVQKVDSKIIEENVPAQYQDKENEWFA

VmFbpA  120 ASGRSRVIAYSTERASAEDIPASVFDLTSEKYQGRFGLAPTNGGF-QSFVTAMRVQHGDE
VvFbpA  119 LTTRTRSVYSSRDRVGKLGDAFTYEDLAKPEFKGKICTRSGKHPYNISLVSAMIAHHGEA
VpFbpA  119 LTLRSRSVYSSRDRVGKLGADFNYADLAKPEFKGKICTRSGKHPYNVSLVSAMIAHNGEA

VmFbpA  179 KTLAWLKAMKANQPK-IYRNNTTQIQAIGDGEIDFALVNNYYLPRFVAA----------N
VvFbpA  179 ETKKWLEGVKANLARKPQGNDRGQVKAIKEGLCDVSLGNSYYLGAMVNDQEQKAWADAVY
VpFbpA  179 ETREWLEGVKANLARKPQGNDRAQVKAIKEGLCDVSLGNSYYLGKMVNDKEQKAWADAVY

VmFbpA  228 ASFPAKQTYFAEGDIGNLVNVAGVAVLKSSKKQPQAIQFIEYMLSPAAQQYFTSVVGEYP
VvFbpA  239 INFPNQQT------TGTHVNISGMAMAKYAPNKANAQKLMEFLTGDKAQHMYAEVNFEYP
VpFbpA  239 INFPNQET------TGTHVNISGMAMAKYSPNKDNAVKLMEFLTGDKAQQMYAEVNYEYP

VmFbpA  288 VTQGIIPNPVLGELDTLLQAAPSIDLDQLAD-LQGTLKLLRDAGL-L
VvFbpA  293 VKAGVERSELVASWGNFK--ADTISLDDIASHHAAAIKLIDEVKFDL
VpFbpA  293 VKEGVKRSELVESWGDFK--ADKLSLDDIAANHEAAIKLLDEVKFDL
```

**Figure. S6.** Sequence alignment of FbpAs from *V. metschnikovii*, *V. vulnificus*, *V. parahaemolyticus*, and *V. cholerae* generated by T-Coffee (version 11.00).

Amino acid residues involved in binding $Fe^{3+}$ are marked by orange boxes. Amino acid residues involved in binding RA are marked by *. Amino acid residues involved in binding RA only are colored blue. Amino acid residues involved in binding both $Fe^{3+}$ and RA are colored red.

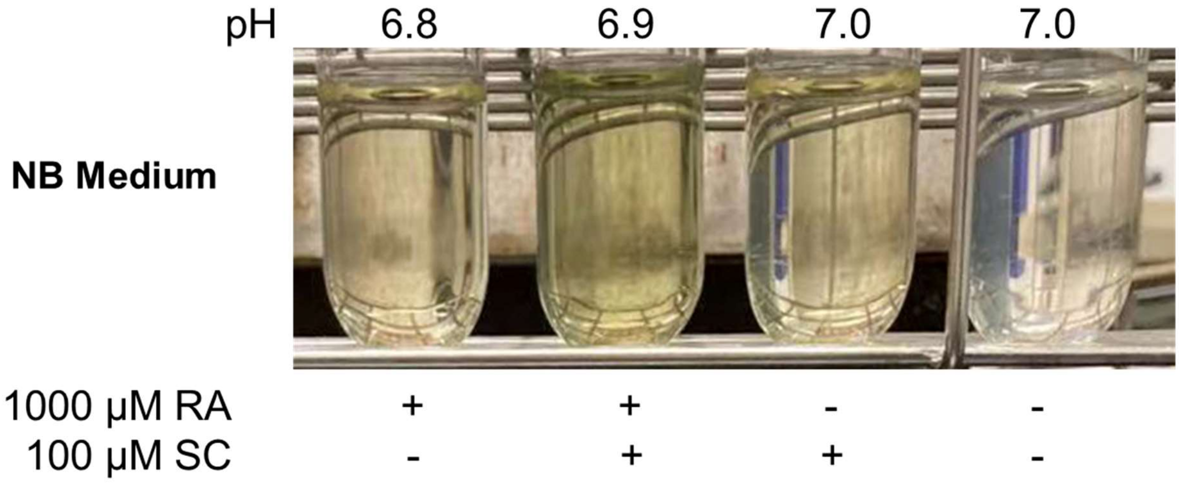


**Figure. S7.** Picture and pH value of NB medium with or without the supplementation of RA/SC.
